# Condensed tannin from *Caragana korshinskii* extraction and protection effects on intestinal barrier function in mice

**DOI:** 10.3389/fvets.2025.1513371

**Published:** 2025-02-03

**Authors:** Xiaoyu Niu, Wei Qu, Zhiyu Chen, Hui Li, Peinan Liu, Mei Sun, Jing Yang, Yuanyuan Xing, Dabiao Li

**Affiliations:** Inner Mongolia Key Laboratory of Animal Nutrition and Feed Science, College of Animal Science, Inner Mongolia Agricultural University, Hohhot, China

**Keywords:** *Caragana korshinskii*, tannins, extraction, intestinal barrier function, antioxidant activities

## Abstract

*Caragana korshinskii* tannins (CKT) were extracted by response surface methodology and the protection effect of CKT on the jejunal mucosal barrier function of mice was investigated. Firstly, this work presents the extraction, purification and characterization of CKT. The results show that the extraction conditions were as follows: extraction temperature was 52°C, extraction time was 95 min, liquid-solid ratio was 20:1 and acetone volume fraction was 62%. The extraction yield of the CKT was 5.34%. The CKT has a typical polyphenol peak with a molecular weight of 8.662 kDa and is composed of epigallocatechin, catechin, epigallocatechin gallate, epicatechin, gallocatechin, epicatechin-3-o-gallate and catechin gallate with a molar ratio of 1:8.88:2.65:1.55:1.92:0.49:0.14. Additionally, the CKT showed strong antioxidants capacity in *vitro*. Secondly, the protection effect of CKT on the growth performance and mucosal barrier function of the mouse jejunum was examined. Totally, sixty KM mice were randomly divided into six treatment groups (*n* = 10) using a single-factor completely randomized experimental design. The treatment groups were intragastrically administered with 0, 25, 50, 100, 200, and 400 mg/kg BW of CKT aqueous solution once a day. The gavage volume was set at 0.2 mL per 10 g of body weight, administered daily for 21 days. The results showed that CKT significantly improved growth performance and physiological state of mouse intestine. CKT strengthened the intestinal physical barrier by upregulating the expression of Occludin and ZO-1 and decreasing the levels of serum diamine oxidase (DAO) and D-lactate (D-LA). Regarding biochemical barrier, CKT could upregulate the activity and gene expression of superoxide dismutase (SOD) and glutathione peroxidase (GSH-Px) and decreasing the content of malondialdehyde (MDA) in jejunum tissues. Generally, CKT may be used as a functional feed additive to regulate intestinal mucosal function, thereby enhancing the health of the intestine and host.

## 1 Introduction

The intestine serves as a strong mucosal barrier, with the mucosa establishing three crucial types of barriers: physical, biochemical, and immunological, that collectively safeguard the overall health of the body (1). Healthy intestinal function promotes nutrient absorption and transport (2). The intestinal physical barrier is a dynamic and permeable barrier composed of single columnar epithelial cells with tight junction, which can effectively prevent the toxic substances in the intestinal cavity from penetrating into the surrounding tissues through the cell space freely (3, 4). Intestinal epithelial cells secrete mucus, antimicrobial peptides, and secretory immunoglobulin, which together constitute a biochemical barrier (5); the intestinal immune barrier is mainly composed of gut-related lymphoid tissue and some antibodies and cytokines secreted by it. These factors can not only reflect the immune state of the intestine, but also regulate the intestinal immune function by regulating the local and systemic immune response of the body (6). However, intestinal mucosa is prone to abnormal stimulation of the external environment to undergo pathological changes, causing harmful metabolites such as lipopolysaccharide (LPS) and histamine and some pathogens to migrate to the circulatory system, causing intestinal microecological imbalance and intestinal tissue morphological changes, leading to the formation of “leaky intestine.”

Natural polyphenols from edible and medicinal plants can influence the inflammatory response and intestinal barrier function, by modulating intestinal epithelial cells, various cytokines, and the mucus secreted by goblet cells (7–10). Extensive studies have suggested that polyphenols like epigallocatechin gallate, resveratrol and tannic acid are able to alleviate oxidative stress and inflammatory response in pigs and poultry (11–14). Research indicates that polyphenols from *Lonicera caerulea L*. can reduce inflammation in mouse models induced by high-fat diets and LPS (15). In addition, Hu et al. (16) found that protocatechuic acid may reduce intestinal inflammation by influencing gut microbiota (17). Most polyphenols are rapidly degraded after ingestion and exhibit low absorption rates; thus, monophenols or phenolic acids metabolized by gut flora are considered the primary bioactive compounds that enter circulation (16, 18, 63). Tannin, as a kind of polyphenol, is a secondary metabolite in plant products with multiple activities such as antioxidant, anti-inflammatory, and anti-bacterial activities, considered potential bioactive metabolites that combat inflammation (19, 20).

*Caragana korshinskii* (CK) is widely cultivated in the arid and semi-arid farming-pasture regions of Northwestern China. Its ecological importance makes it a popular choice as a shrub species for afforestation efforts and desertification prevention (21). As a forage bush, CK is rich in phenolic acids (tannins, syringic acid, gallic acid, etc.), protein, mineral elements, and a variety of nonessential and essential amino acids, so its ingestion would directly interact with the intestinal mucosa (22, 23). Our recent study revealed that *Caragana korshinskii* tannin (CKT) improved serum immunity and antioxidant capacity by modulating enzyme activity and cytokine content, and reduced CH_4_ emission by modulating the abundance of ruminal methanogens in sheep (24, 25, 64). Based on our previous study and literatures, we hypothesized that improving intestinal barrier function is a potential mechanism through which CKT exert their protection effects. Hence, in this study, CKT were extracted by response surface methodology and the effect of CKT on the jejunal mucosal barrier function of mice was investigated using an oral administration system.

## 2 Materials and methods

### 2.1 Extraction, optimization and structural analysis of CKT

#### 2.1.1 Preparation of CKT

CK was collected from Siziwang Banner (Inner Mongolia, China) in June. The CKT was prepared using an organic solvent extraction method. First, the whole CK was washed and dried. The dried CK was then ground into powder. This powder underwent two extractions with petroleum ether for 2 h. After drying at 60°C for 12 h, a 20 g defatted sample was extracted under varying conditions: extraction temperature (30–70°C), extraction time (40–120 min), liquid-solid ratio (10–50 mL/g), and acetone volume fraction (40%−80%). The resulting solution was lyophilized using a vacuum freeze dryer. CT from CK were purified and used as standards to measure the concentration of CT in crude extracts according to the procedure of Palacios et al. (26).

#### 2.1.2 Single factor evaluation

Extraction temperature, time, liquid-solid ratio, and acetone volume fraction were established to assess the effects on CKT yield. Five treatments were conducted, and each with three replicates for every factor.

#### 2.1.3 Response surface methodology optimization

Four factors, namely *X*_1_ (extraction temperature, °C), *X*_2_ (extraction time, min), *X*_3_ (liquid–solid ratio, mL/g), and *X*_4_ (acetone volume fraction, %) were determined their influences on CKT yield. According to Box-Behnken design (BBD) method, three levels were designed, namely *X*_1_ (50°C, 60°C, 70°C), *X*_2_ (60 min, 80 min, 100 min), *X*_3_ (20:1, 30:1, 40:1), and *X*_4_ (60%, 70%, 80%). The design of each factor and level was shown in Table 1, the whole design consisted of 29 experiments was shown in Table 2.

**Table 1:** Factors and levels design in the Box-Behnken design.

**Factors**	**Levels**		
	−**1**	**0**	**1**
Extraction temperature (°C)	50	60	70
Extraction time (min)	60	80	100
Liquid–solid ratio (mL/g)	20:1	30:1	40:1
Acetone volume fraction (%)	60	70	80

**Table 2:** BBD with four variables and results for the yield of CKT.

**Run**	***X*_1_ (extraction temperature, °C)**	***X*_2_ (extraction time, min)**	***X*_3_ (liquid-solid ratio, mL/g)**	***X*_4_ (acetone volume fraction, %)**	**Y (CKT yield, mg/g)**
1	0	0	0	0	41.8
2	0	0	0	0	39.6
3	0	0	1	1	27.0
4	1	−1	0	0	30.9
5	0	0	1	−1	38.3
6	−1	0	1	0	38.0
7	0	−1	−1	0	33.1
8	−1	−1	0	0	36.1
9	0	1	1	0	32.7
10	1	0	1	0	21.1
11	0	0	−1	1	31.8
12	0	0	0	0	39.0
13	0	0	−1	−1	46.8
14	−1	0	0	−1	36.9
15	0	−1	0	1	24.6
16	0	1	−1	0	48.1
17	0	0	0	0	37.6
18	−1	0	−1	0	37.0
19	1	0	0	−1	26.5
20	0	1	0	−1	49.9
21	−1	1	0	0	47.2
22	0	−1	0	−1	33.6
23	1	0	0	1	21.5
24	0	1	0	1	27.8
25	0	−1	1	0	34.8
26	−1	0	0	1	29.8
27	1	0	−1	0	24.6
28	0	0	0	0	36.7
29	1	1	0	0	28.0

A second-order polynomial model was proposed for the response (*Y*_*i*_), and the regression coefficients were as follows:


(1) $$\begin{array}{c} Y_{i}=\beta_{0}+\overset{4}{\underset{i=1}{\sum }}\beta_{i}X_{i}+\overset{4}{\underset{i=1}{\sum }}\beta_{ii}X_{i}^{2}+\overset{3}{\underset{i=1}{\sum }}\overset{4}{\underset{j=i+1}{\sum }}\beta_{ii}X_{i}X_{j} \end{array}$$

where *Y*_*i*_ is the predicted response value, β_0_ is the model constant, β_*i*_ represents the linear effect terms, β_*ii*_ is the quadratic effect and β_*ij*_ is the interaction effect of the variables.

#### 2.1.4 Analysis of tannin monomer composition

The CKT sample (0.1 g) was dissolved in 2.5 M HCl at 75°C for 2.5 h. The supernatant of 200 μL was then evaporated. For UHPLC–ESI–MS/MS analysis, the resulting sample was re-dissolved in 0.2 mL of 50% methanol. The analytical conditions were as follows: UPLC column: Waters ACQUITY UPLC HSS T3 (1.7 μm, 2.0 mm × 48 mm); column temperature: 39°C; flow rate: 0.28 mL/min; injection volume: 1.8 μL; gradient program: 90:10 V/V at 0 min, 90:10 V/V at 1.2 min, 60:30 V/V at 5.5 min, 5:90 V/V at 7.5 min, 5:90 V/V at 9 min, 95:15 V/V at 9.5 min, and 95:11 V/V at 9 min. Catechin, epicatechin, gallocatechin, epigallocatechin, ellagic acid, epicatechin-3-o-gallate, gallocatechin gallate, epigallocatechin gallate, and catechin gallate were used as the monomer standards.

### 2.2 Antioxidative activity assay for CKT

#### 2.2.1 Assay of DPPH free radical scavenging activity

The ability of CKT to scavenge DPPH hydroxyl radicals was assessed using a slightly modified method from Di (27). Briefly, various concentrations of CKT (0.0, 0.2, 0.4, 0.6, 0.8, and 1.0 mg/mL) were mixed with ethanol containing DPPH. The reaction solution was incubated in the dark at 26°C for 28 min, after which the absorbance was measured at 520 nm. Vitamin C (Vc) was used as a positive control. The scavenging rate was calculated according to the formula:


(2) $$\begin{array}{c} Scavenging\;rate\;of\;CKT\;\left(\%\right)=\frac{\left[A_{0}-\left(A_{1}-A_{2}\right)\right]}{A_{0}}\times 100\% \end{array}$$

Where *A*_1_ is the absorbance of CKT sample, *A*_2_ is the absorbance of the sample under conditions as *A*_1_ with distilled water instead of DPPH, and *A*_0_ is the absorbance of the distilled water instead of sample.

#### 2.2.2 Assay of ABTS free radical scavenging activity

Following Re's description, 200 μL of CKT solution was mixed with 4.6 mL of diluted ABTS solution and allowed to react for 7 min at 26°C. The absorbance was then measured at 730 nm. Prior to use, ABTS solution was diluted with a phosphate buffer solution (pH 7.4, 0.2 M) to achieve an absorbance of 0.70 ± 0.01 at 734 nm (28). The scavenging rate was calculated using the same formula as above.

#### 2.2.3 Assay of hydroxyl radical scavenging activity

CKT solution (1.2 mL) were mixed with FeSO_4_ (1 mL, 9 mM), H_2_O_2_ (1 mL, 9 mM), and salicylic acid (1 mL, 9 mM), respectively, and the absorbance at 524 nm was measured after reaction at 38°C for 25 min (29). The scavenging rate was calculated using the same formula as above.

#### 2.2.4 Determination of reducing power

The reducing power of CKT was performed by spectroscopic method (30). The CKT solution (2.0 mL) of different concentrations was blended with PBS (2.5 mL, 200 mM, pH 6.4) and K_3_[Fe(CN)_6_] (1.6 mL, 1% w/v), respectively. After incubation for 18 min at 54°C, trichloroacetic acid (2.8 mL, 12% w/v) was added to stop the reaction. The mixture was then centrifuged at 1,600 g for 8 min. The supernatant was subsequently combined with distilled water and ferric chloride. After 8 min, the absorbance of the solution was measured at 705 nm, with an increase in absorbance indicating a greater reducing power.

### 2.3 Animals and experimental design

A total of 60 male KM mice, aged 4–6 weeks, were purchased from the Laboratory Animal Center of Inner Mongolia University (Hohhot, China). Mice were randomly divided into 6 groups (*n* = 10). Group 1 was given distilled water as a control group (CON), groups 2, 3, 4, 5, and 6 were administered 25, 50, 100, 200, and 400 mg/kg of CKT, respectively. The mice were kept in individually ventilated cages (IVC) and had access *ad libitum* to autoclaved rodent chow (Ssnif, Soest, Germany) and acidifed water, all cages were located in the same IVC rack during the whole period of the experiment and were housed under 12:12 h light/dark cycles, at a 22 ± 2°C room temperature and 55 ± 5% humidity, Six mice were housed together in one cage. CKT was dissolved in 0.9% saline solution. The gavage volume was set at 0.2 mL per 10 g of body weight, administered daily for 21 days. Twelve h after the final gavage, the jejunum was collected from the mice. The lengths were measured, and the segments were washed in HBSS. Approximately 1 cm of each segment was placed in 4% paraformaldehyde for 12 h for histological analysis, while the remaining intestinal segments were stored at −80°C for future use.

### 2.4 Determination of growth performance

The mice were weighed on day 1 and day 21. The total feed consumption and the total weight of mice for each cage were separately recorded to calculate average daily gain (ADG), average feed daily intake (ADFI), and the ratio of feed to gain (FCR).

### 2.5 Histomorphological observation

Jejunum tissues were fixed in 4% paraformaldehyde for 12 h, then embedded in paraffin at 65°C. Sections were cut at a thickness of 5 μm using a microtome and subsequently stained with hematoxylin and eosin (H&E) for 10 min. Using a light microscope, its profile was meticulously observed and photographed. Both villus height (VH) and crypt depth (CD) were measured using a optical microscope (Nikon Eclipse, E100) and imaging system (NIKON DS-U3).

### 2.6 Determination of intestinal permeability markers in blood

In this study, the activity of DAO was measured using a commercial kit (Nanjing Jiancheng Bioengineering Institute, AO88-1-1). D-LA levels in serum was determined using ELISA kits (MEIMIAN, MM-0369).

### 2.7 Determination of MDA concentrations and activities of SOD, GSH-Px, and CAT

MDA concentrations, along with the enzymatic activities of SOD, GSH-Px, and CAT, were measured according to the method described in previous studies (31, 32) using commercially available kits (Nanjing Jiancheng Bioengineering Institute, Nanjing, China).

### 2.8 Quantitative real-time polymerase chain reaction

Total RNA was isolated from each jejunum segment using TRIzol, and cDNA was synthesized. SYBR Premix ExTaq™ was employed to amplify and analyze the cDNA fragments. qRT-PCR was conducted using SYBR Select Master Mix (TaKaRa, Dalian, China). The PCR conditions were as follows: a cycle of initial denaturing at 95°C for 30, followed by 40 cycles of 95°C for 5 s, 59–68°C for 30 s, follow by 72°C for 30 s. Relative mRNA levels were calculated using the comparative CT method with the formula 2^−ΔΔCt^, with the β-actin gene serving as the reference to normalize the expression of target genes (33). The primer sequences used are presented in Table 3.

**Table 3:** qRT-PCR primers sequence list.

**Gene name**	**Primer**	**Sequence (5^′^−3^′^)**	**Gene Bank No**.	**Product length**
SOD	Forward	TGTCCATTGAAGATCGTGTGAT	NM_001145185.2	85
	Reverse	TCATCTTGTTTCTCATGGACCA		
GSH-Px	Forward	GTTTGAGAAGTGCGAAGTGAAT	XM_004018462.5	130
	Reverse	CCCCTCCTCGTTCAAGATGT		
CAT	Forward	AATCGGACGGCAATAGGAGT	XM_060400055.1	194
	Reverse	CCATAATCCGGATCTTCCTG		
Claudin	Forward	GGGGACAACATCGTGACCG	NM_001244539	200
	Reverse	AGGAGTCGAAGACTTTGCACT		
ZO−1	Forward	GCCGCTAAGAGCACAGCAA	XM_003121672	182
	Reverse	TCCCCACTCTGAAAATGAGGA		
Occludin	Forward	TTGAAAGTCCACCTCCTTACAGA	NM_001163647	104
	Reverse	CCGGATAAAAAGAGTACGCTGG		
β-actin	Forward	GGCTGTATTCCCCTCCATCG	NM_007393.5	154
	Reverse	CCAGTTGGTAACAATGCCATGT		

### 2.9 Western blot analysis

The method for protein extract preparation and western blotting were the same as our previous study (25). The jejunum segment tissue (100 mg) was taken and added 850 μL of lysate-protease inhibitor (100:1) mixture in a tissue grinder and homogenized thoroughly, centrifuged at 4°C, 12,000 rpm for 12 min. Afterward, protein concentration was determined by BCA kit (Termo Fisher Scientifc, USA), and the total protein was denatured for 4 min at 100°C. Protein samples were separated by SDS-PAGE gels and transferred to PVDF membrance. The membrane were blocked with 5% fat-free milk for 2.5 h. After completing, it was washed with TBST four times and incubated in Claudin, Occludin, ZO-1 and β-actin (1:1,000) antibodies overnight at 4°C, respectively. The strips were placed into the corresponding secondary antibodies (1:10,000) and incubated at room temperature for 2.5 h. It was followed by four washes and scanning of the membranes using an Odyssey Infrared Imaging System (Odyssey Clx, LI-COR Biosciences, Lincoln, NE).

### 2.10 Statistical analysis

In order to determine the effect of treatments on growth performance, jejunum morphology, jejunum permeability, jejunum antioxidant capacity and the genes expression, each mice was considered as an experiment unit. The normality of the distribution of variables was tested by the Shapiro - Wilk test and all variables were normally distributed. All results of 6 dietary treatment groups were subjected to ANOVA where diets were treated as the fixed effect, animal were considered as the random effect using the GLM procedure of SAS (SAS Inst. Inc., Cary, NC, USA; version 9.0). When significant differences were observed, Tukey's test was used to adjust for multiple comparisons. The PROC MIXED model was used including random and fixed effects as follows: Yij = μ + Li + Tj + εij, where Yij is the dependent variable, μ is the overall mean, Li is the random effects of mice (*i* = 10), Tj is the fixed effect of CKT supplements (j = 0, 25 mg/kg, 50 mg/kg, 100 mg/kg, 200 mg/kg and 400 mg/kg), and εij is the error term. Variability in the data was expressed as the standard error means and a probability level of *P* < 0.05 was considered statistically significant (25).

## 3 Results

### 3.1 Optimization of CKT extraction

#### 3.1.1 Effects of extraction temperature on the yield of CKT

**Figure 1:**
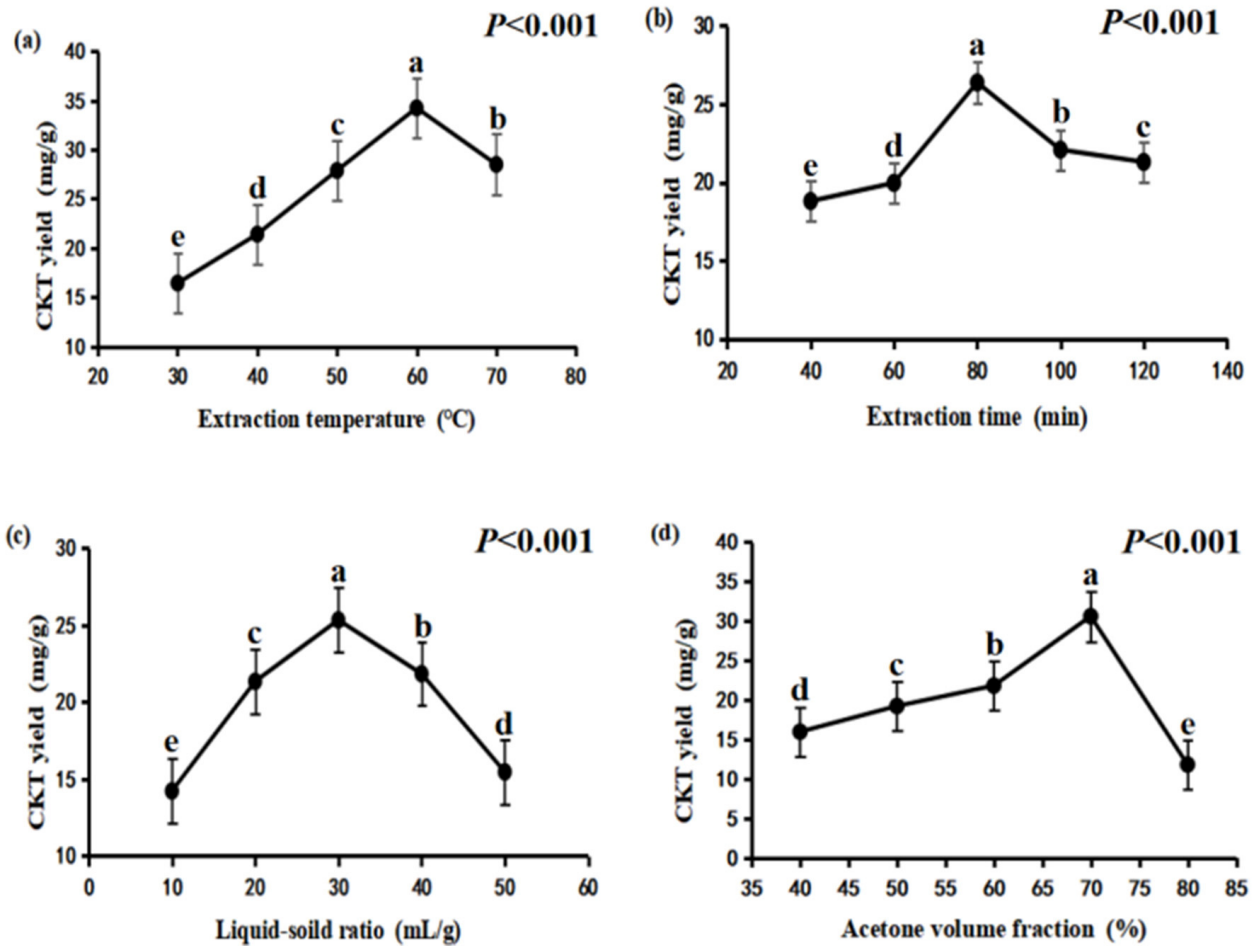
Effects of different extraction conditions onCKT yield. **(A)** extraction temperature; **(B)** extraction time; **(C)** liquid–solid ratio; **(D)** acetone volume fraction. CKT, *Caragana korshinskii* tannin.

The experimental conditions for evaluating the effect of extraction temperature were set with an extraction time of 80 min, a liquid-to-solid ratio of 30:1, and an acetone volume fraction of 60%. As shown in Figure 1A, the extraction yield of CKT rose as the temperature increased from 30°C to 60°C, but then decreased when raised from 60°C to 70°C. This is because a higher extraction temperature promotes the mass transfer rate of CT molecules and improves CT solubility (34). However, excessive temperatures may degrade thermo-sensitive compounds like CT, leading to a reduced yield.

#### 3.1.2 Effects of extraction time on the yield of CKT

The experimental conditions for the single factor change of extraction time was an extraction temperature of 50°C, liquid-soild ratio of 30:1 and acetone volume fraction of 60%. CKT yield increased first and then decreased, the maximum CT yield was obtained at extraction time of 80 min (Figure 1B).

#### 3.1.3 Effects of liquid-soild ratio on the yield of CKT

Liquid-soild ratio was important factor that affected tannin extraction yield, additionally, excess use of acetone will lead to waste. In the present study, the experimental conditions for the single factor change of liquid-soild ratio was an extraction temperature of 50°C, extraction time of 80 min and acetone volume fraction of 60%. As indicated in Figure 1C, the relatively lower tannin yield was obtained at liquid-solid ratio of 10:1, which likely occurs due to solvent saturation (35). The highest yield of tannins was achieved at a liquid-to-solid ratio of 30:1; however, the extraction yield of CKT declined when the ratio was increased from 30:1 to 50:1.

#### 3.1.4 Effects of acetone volume fraction on the yield of CKT

The experimental conditions for the single factor change of the acetone volume fraction was an extraction temperature of 50°C, extraction time of 80 min and liquid-soild ratio of 30:1. Acetone volume fraction of 70% led to the highest CKT yield as demonstrated in Figure 1D. But the extraction rate of CKT decreased sharply when the acetone volume fraction exceeded 70%.

According to the single-factor study, extraction temperature of 50–70°C, extraction time of 60–100 min, liquid–solid ratio of 20:1–40:1 and acetone volume fraction of 60%−80% were adopted for the RSM experiments.

### 3.2 BBD and RSM optimization

The results from the RSM experiments are presented in Table 4. By conducting multiple regression analysis on the experimental data, a predictive model was formulated, represented by the following second-order polynomial equation based on the coded values: *Y* = 38.94 – 6.02*X*_1_ + 3.39*X*_2_– 2.46*X*_3_– 5.81*X*_4_– 3.50*X*_1_*X*_2_– 1.15*X*_1_*X*_3_ + 0.51*X*_1_*X*_4_– 4.30*X*_2_*X*_3_– 3.30*X*_2_*X*_4_ + 0.92*X*_3_*X*_4_– 5.86$X_{1}^{2}$ + 0.28$X_{2}^{2}$- 1.40$X_{3}^{2}$- 3.75$X_{4}^{2}$, where Y denotes CKT extraction yield (mg/g), *X*_1_ denotes the extraction temperature (°C), *X*_2_ denotes the extraction time (min), *X*_3_ denotes the liquid-solid ratio (mL/g), and *X*_4_ denotes the acetone volume fraction (%). For the quadratic regression model, R^2^ was 0.9041, suggesting that the model accounted for 90.41% of the variance in CKT yield, demonstrating high accuracy and reliability (36). The lack of fit was not significant, indicating that the model was stable and adequately applicable for predicting extraction rates within the experimental range. Additionally, the model's significance (Table 4) suggests it effectively captures the interactions between variables (37). From Table 4, the linear coefficients (*X*_1_, *X*_2_, and *X*_4_), interaction coefficients (*X*_2_ and *X*_3_), and quadratic coefficients ($X_{1}^{2}$ and $X_{4}^{2}$) of the model were of great significance, and their *p*-values were < 0.05. Moreover, by analyzing the quadratic and linear coefficients, the order of factors influencing the extraction ratio of CKT was determined as follows: extraction temperature > acetone volume fraction > extraction time > liquid-solid ratio.

**Table 4:** Analysis of variance of the regression model.

**Source**	**Sum of squares**	**df**	**Mean square**	***F-*value**	***P-*value**
Model	1,520.32	14	108.59	8.72	0.0001
A	435.49	1	435.49	34.96	< 0.0001
B	138.31	1	138.31	11.10	0.0049
C	72.82	1	72.82	5.85	0.0298
D	405.65	1	405.65	32.57	< 0.0001
AB	48.86	1	48.86	3.92	0.0676
AC	5.31	1	5.31	0.43	0.5243
AD	1.04	1	1.04	0.08	0.7768
BC	73.79	1	73.79	5.92	0.0289
BD	43.43	1	43.43	3.49	0.0829
CD	3.40	1	3.40	0.27	0.6093
A^2^	222.79	1	222.79	17.89	0.0008
B^2^	0.49	1	0.49	0.04	0.8452
C^2^	12.70	1	12.70	1.02	0.3297
D^2^	91.12	1	91.12	7.32	0.0171
Residual	174.37	14	12.46		
Lack of fit	159.03	10	15.90	4.15	0.0914
Pure error	15.34	4	3.84		
Cor total	1,694.7	28			
R^2^	0.90				

**Figure 2:**
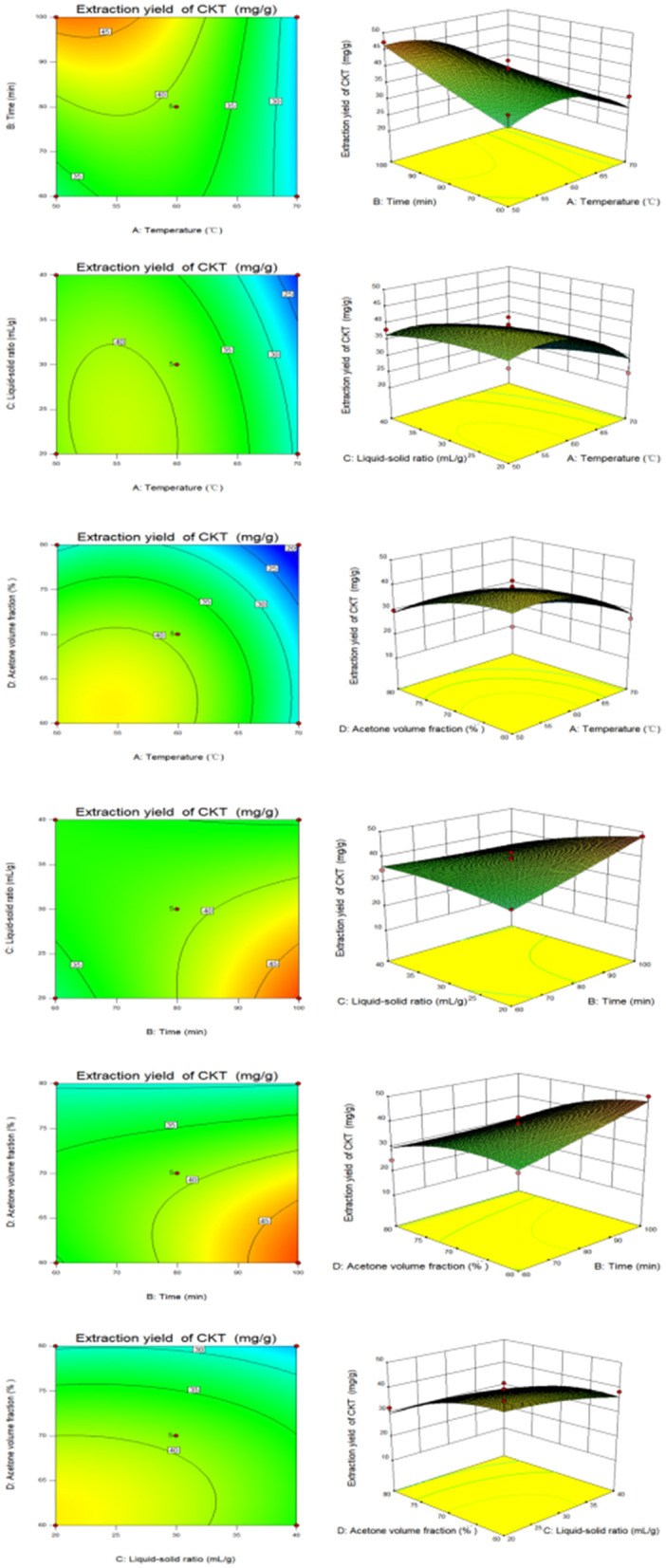
The response surface plots and contour plots between the factors and CKT yield. CKT, *Caragana korshinskii* tannin.

Based on the data from the response surface method analysis, Figure 2 presents the response surface and contour plots illustrating the relationship between the factors and CKT yield. These plots depict the interactions between two variables while maintaining the other variables at their zero levels for CKT yield (38). As shown in Figure 2, the interaction of extraction time and liquid-solid ratio had a significant effect on CKT yield. All drawings can be explained similarly.

The optimum extraction conditions of CKT were as follows: extraction temperature of 51.84°C, extraction time of 95.46 min, liquid-solid ratio of 20.23:1 and acetone volume fraction of 62.48%. The prediction yield of CKT was estimated to be up to 5.35%. Considering to the feasibility of the actual mechanical process, the extraction conditions of the CKT were modified to extraction temperature of 52°C, extraction time of 95 min, liquid-solid ratio of 20:1 and acetone volume fraction of 62%. With this understanding, three parallel experiments were conducted, and the average CKT yield was found to be 5.34%. This value closely aligned with the theoretical prediction, demonstrating that using this model for optimizing the extraction process of CKT is practically significant.

### 3.3 Preliminary characterization of CKT

**Figure 3:**
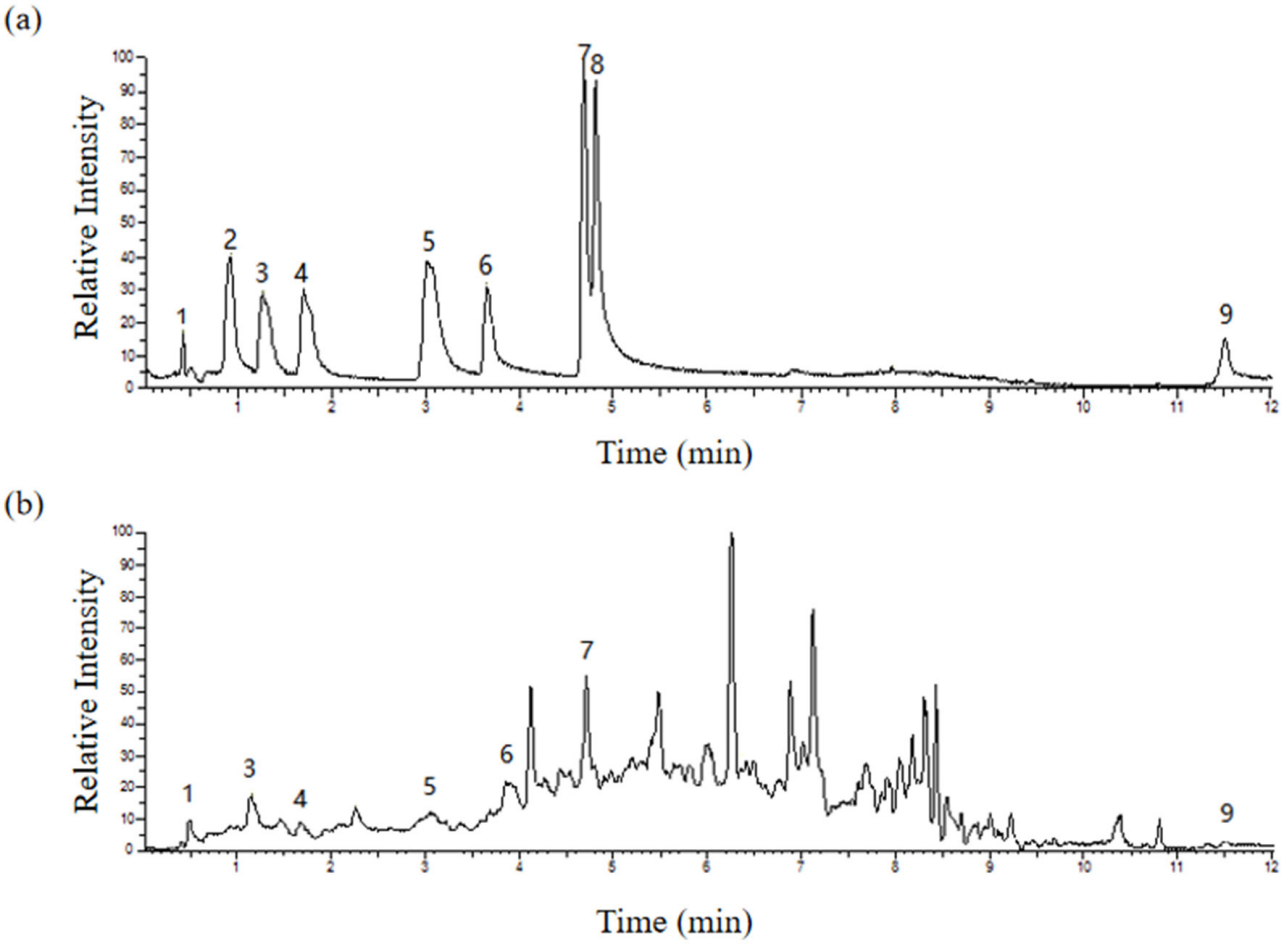
HPLC traces of the standard monomer of **(A)** CT and **(B)** CKT. Peak: 1-Epigallocatechin, 3-Gallocatechin, 4-Epicatechin-3-o-gallate, 5-Epicatechin, 6-Epigallocatechin gallate, 7-Catechin, and 9-Catechin gallate. CT, condensed tannin. CKT, *Caragana korshinskii* tannin.

The monomer composition of the CKT is illustrated in Figure 3. The CKT has a typical polyphenol peak with a molecular weight of 8.662 kDa and is composed primarily of epigallocatechin: catechin: epigallocatechin gallate: epicatechin: gallocatechin: epicatechin-3-o-gallate: catechin gallate = 1:8.88:2.65:1.55: 1.92:0.49:0.14. The molar ratio of tannin monomer is illustrated in Table 5.

**Table 5:** The tannin monomer molar ratio of CKT.

**Compounds**	**Molar ratio (%)**
Epigallocatechin	6.01
Catechin	53.37
Epigallocatechin gallate	15.94
Epicatechin	9.30
Gallocatechin	11.57
Epicatechin-3-o-gallate	2.97
Catechin gallate	0.84

### 3.4 Antioxidant activity assays

#### 3.4.1 Scavenging effect of CKT on DPPH radicals

**Figure 4:**
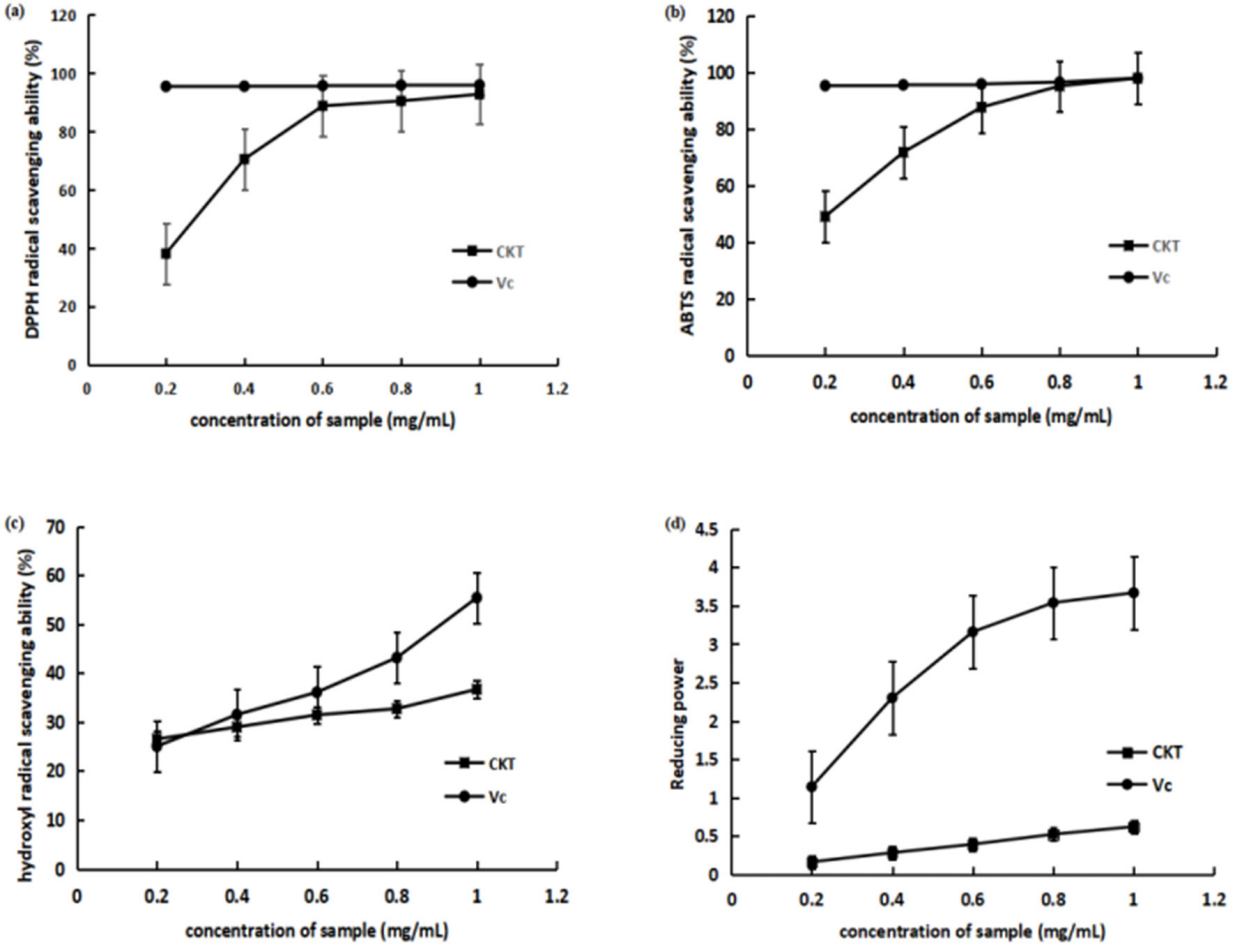
Antioxidant activity of CKT *in vitro*. **(A)** DPPH radical scavenging activity; **(B)** ABTS radical scavenging activity; **(C)** hydroxyl radical scavenging activity; **(D)** reducing power. CKT, *Caragana korshinskii* tannin.

As shown in Figure 4A, CKT exhibited lower DPPH radical scavenging activity compared to vitamin C (Vc) across all tested concentrations, although the scavenging capacity of CKT increased in a dose-dependent manner. At a concentration of 1 mg/mL, the scavenging rate reached 92.70%.

#### 3.4.2 Scavenging effect of CKT on ABTS radicals

As illustrated in Figure 4B, compared to Vc, CKT demonstrated lower ABTS radical scavenging activity across all tested concentrations. At the concentration of CKT was 1 mg/mL, its ABTS radical scavenging rate reached 97.91%. The IC_50_ of the scavenging ability of CKT on ABTS radical was 0.21 mg/mL. The results indicated that CKT has a noticeable ABTS radical scavenging activity.

#### 3.4.3 Scavenging effect of CKT on hydroxyl radicals

The scavenging effect on hydroxyl radical of CKT was shown in Figure 4C. At a concentration of 1 mg/mL, the hydroxyl radical scavenging activity of CKT was 36.67%. The IC_50_ of the scavenging ability of CKT on hydroxyl radical was 0.19 mg/mL, which suggested a satisfying radical scavenging activity.

#### 3.4.4 Reducing power of CKT

The reducing power of CKT was assessed, with results displayed in Figure 4D. At a concentration of 1.0 mg/mL, CKT demonstrated a reducing ability of 0.62. As the concentration of CKT increased, a notable upward trend was observed.

### 3.5 Effects of CKT on growth performance

As shown in Table 6, with the increase of CKT concentration, the ADG increased quadratically (*P*_quadratic_ < 0.05), with the highest value at 25 mg/kg CKT (*P* < 0.05). Supplementation of 25, 50, and 100 mg/kg CKT significantly increased (*P* < 0.05) the final BW of mice. Meanwhile, a significant increase (*P* < 0.05) in the ADG was observed in the 25 mg/kg CKT group, as compared with the control group. However, dietary treatments showed a limited effect on ADFI and FCR of mice (*P* > 0.05).

**Table 6:** Effects of CKT on growth performance in mice.

**Items**	**Dietary CKT levels (mg/kg BW)**						**SEM**	***P*** **-Value**		
	**0**	**25**	**50**	**100**	**200**	**400**		**ANOVA**	**Linear**	**Quadratic**
Intial BW, g	22.05	23.35	23.40	22.99	22.98	22.52	1.010	0.356	0.786	0.632
Final BW, g	24.95^c^	29.75^a^	28.73^ab^	27.51^ab^	26.17^bc^	26.12^bc^	0.961	< 0.001	0.330	0.519
ADG, g	0.15^b^	0.30^a^	0.24^ab^	0.25^ab^	0.23^ab^	0.17^b^	0.025	0.047	0.541	0.004
ADFI, g	0.26	0.28	0.31	0.32	0.23	0.19	0.035	0.162	0.105	0.269
FCR	1.49	1.55	1.43	1.46	1.48	1.39	0.145	0.999	0.749	0.951

CKT, *Caragana korshinskii* tannin; BW, body weight; ADG, average daily gain; ADFI, average daily feed intake; FCR, feed conversion ratio; ^a,b,c^means with different letters differ significantly (*P* < 0.05); SEM, standard error of mean.

### 3.6 Effects of CKT on jejunum morphology

**Figure 5:**
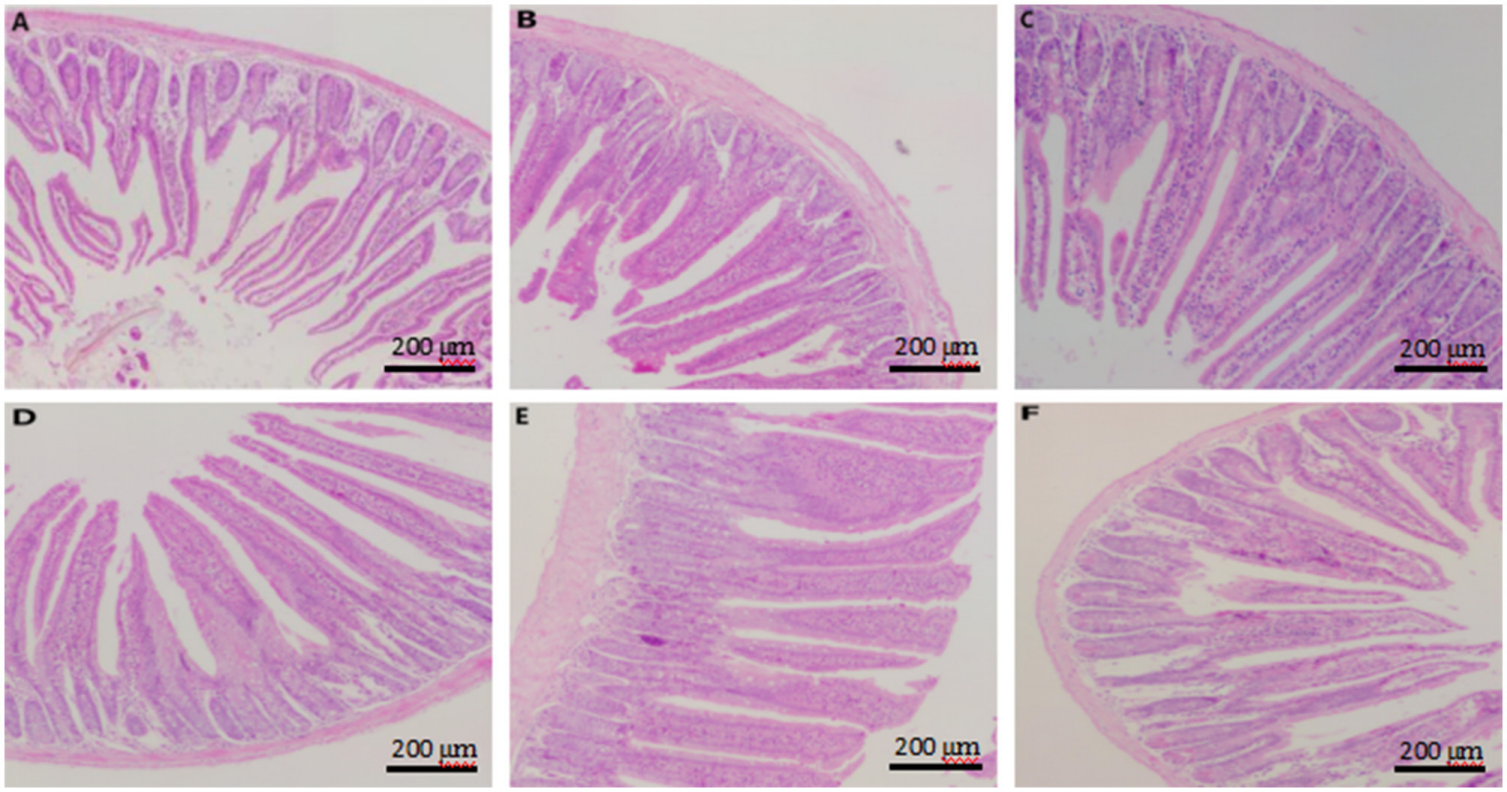
Effect of dietary supplementation with CKT on jejunum morphology in mice. **(A–F)** Represent control, 25 mg/kg CKT, 50 mg/kg CKT, 100 mg/kg CKT, 200 mg/kg CKT, and 400 mg/kg CKT, respectively. CKT, *Caragana korshinskii* tannin.

The results of analysis of jejunum morphology were listed in Table 7 and Figures 5A–F. The results indicated that, with the increase of CKT concentration, the jejunum VH increased quadratically (*P*_quadratic_ < 0.05), with the highest value at 100 mg/kg CKT (*P* < 0.05). In comparison to the control group, supplementation of 25, 50, 100, and 200 mg/kg CKT significantly increased (*P* < 0.05) jejunum VH and VH:CD of mice. It is worth mentioning that, compared with other groups, a significant increase (*P* < 0.05) in the VH and VH:CD was observed in the 100 mg/kg CKT group. However, VH and VH:CD were not affected by 400 mg/kg CKT inclusion (*P* > 0.05). Moreover, CD did not differ significantly among the various treatments (*P* > 0.05).

**Table 7:** Effects of dietary supplementation with CKT on jejunum morphology in mice.

**Items**	**Dietary CKT levels (mg/kg BW)**						**SEM**	***P*** **-Value**		
	**0**	**25**	**50**	**100**	**200**	**400**		**ANOVA**	**Linear**	**Quadratic**
VH, μm	290.35^d^	360.27^bc^	420.47^b^	510.94^a^	410.71^b^	316.51^cd^	9.991	0.001	0.036	0.011
CD, μm	103.47	103.83	109.35	118.65	112.85	98.80	6.721	0.557	0.782	0.625
VH:CD	2.81^d^	3.564^bc^	3.954^b^	4.754^a^	4.051^b^	3.145^cd^	0.103	0.015	0.052	0.068

CKT, *Caragana korshinskii* tannin; VH, villus height; CD, crypt depth; ^a,b,c,d^means with different letters differ significantly (*P* < 0.05); SEM, standard error of mean.

As showed in Figure 5, jejunum villus of control mice showed atrophied villus and disorderly epithelium (Figure 5A), the jejunum morphology of 400 mg/kg CKT mice were as atrophied as mice in the control group (Figure 5F), whereas 100 and 200 mg/kg CKT mice showed orderly arranged intestinal epithelial cells and smooth villus border (Figures 5D, E). Hardly protective effect could be observed in 25 and 50 mg/kg CKT mice (Figures 5B, C), as shown by the discontinuous villus.

### 3.7 Effects of CKT on intestinal permeability

To further investigate the structural integrity and physical barrier function of the jejunum tissues, DAO and D-Lactic acid were determined by enzyme-linked immunosorbent assay (Table 8). With the increase of CKT concentration, both the content of D-Lactic acid an DAO were decreased linearly (*P*_linear_ < 0.05) and quadratically (*P*_quadratic_ < 0.05), with the lowest value at 100 mg/kg CKT (*P* < 0.01). Dietary CKT supplementation significantly decreased (*P* < 0.05) the D-Lactic acid content when compared with the control group. The addition of 100 mg/kg CKT treatment significantly decreased (*P* < 0.05) the jejunum DAO content when compared with the control group.

**Table 8:** Effects of dietary supplementation with CKT on jejunum markers of intestinal permeability in mice.

**Items**	**Dietary CKT levels (mg/kg BW)**						**SEM**	***P*** **-Value**		
	**0**	**25**	**50**	**100**	**200**	**400**		**ANOVA**	**Linear**	**Quadratic**
DAO, U/mL	22.99^a^	23.82^a^	23.02^a^	19.78^b^	21.77^ab^	24.12^a^	0.850	0.025	0.044	0.004
D-LA, ng/mL	14.30^a^	13.25^b^	13.45^b^	9.80^d^	11.57^c^	13.58^b^	0.262	< 0.001	0.006	< 0.001

CKT, *Caragana korshinskii* tannin; DAO, diamine oxidase; D-LA, D-lactic acid; ^a,b,c,d^means with different letters differ significantly (*P* < 0.05); SEM = standard error of mean.

### 3.8 Effects of CKT on antioxidant capacity of jejunum tissues

**Figure 6:**
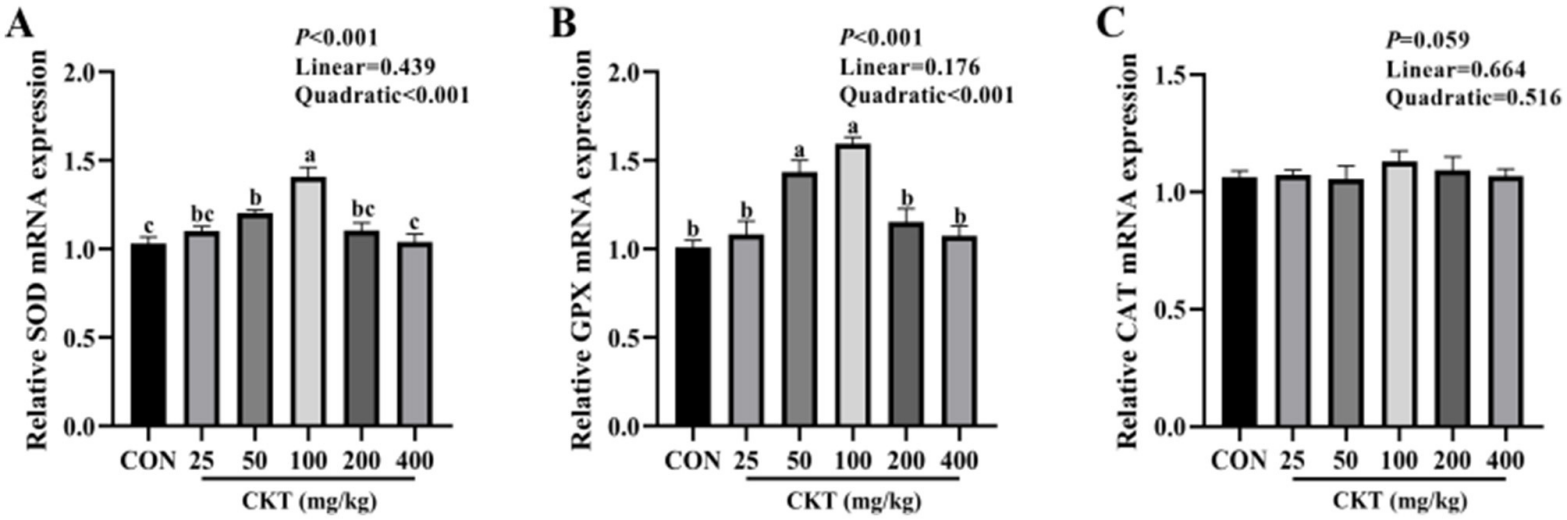
Effect of dietary supplementation with CKT on antioxidant gene expression of jejunum tissues in mice. SOD, superoxide dismutase; GSH-Px, glutathione peroxidase; CAT, catalase; CKT, *Caragana korshinskii* tannin. **(A)** Relative SOD mRNA expression, **(B)** Relative GPX mRNA expression, **(C)** Relative CAT mRNA expression.

To verify whether CKT ameliorates stress by activating the antioxidant system, we investigated the activity and gene expression of antioxidant enzymes, as well as the levels of oxidative products (Table 9, Figure 6). With the increase of CKT concentration, both the activity and gene expression of SOD and GSH-Px were increased quadratically (*P*_quadratic_ < 0.05), with the highest value at 100 mg/kg CKT (*P* < 0.05), however, the MDA concentration was decreased linearly (*P*_linear_ < 0.05) and quadratically (*P*_quadratic_ < 0.05), with the lowest value at 100 mg/kg CKT (*P* < 0.01). In addition, the results showed that the addition of 100 mg/kg CKT significantly increased (*P* < 0.05) the SOD and GSH-Px activities of jejunum tissues in mice. Moreover, the addition of 100 mg/kg CKT significantly decreased (*P* < 0.05) MDA concentration. However, there were no significant difference in the CAT activities between CON group and CKT groups (*P* < 0.05). In addition, 50 mg/kg and 100 mg/kg CKT treatment significantly increased (*P* < 0.05) the mRNA expression level of SOD and GSH-Px in jejunum when compared with the control group (Figures 6A, B). Meanwhile, a significant increase (*P* < 0.05) in the mRNA expression of SOD was observed in the 100 mg/kg CKT group, as compared with the 50 mg/kg CKT group (Figure 6A). However, dietary treatments showed a limited effect on the CAT mRNA expression of jejunum tissues in mice (*P* > 0.05) (Figure 6C).

**Table 9:** Effects of dietary supplementation with CKT on antioxidant capacity of jejunum tissues in mice.

**Items**	**Dietary CKT levels (mg/kg BW)**						**SEM**	***P*** **-Value**		
	**0**	**25**	**50**	**100**	**200**	**400**		**ANOVA**	**Linear**	**Quadratic**
SOD, U/mg protein	6.89^b^	7.33^b^	7.46^b^	8.88^a^	7.67^b^	7.40^b^	0.357	0.002	0.150	0.001
GSH-Px, U/mg protein	14.47^b^	15.34^b^	15.35^b^	17.08^a^	14.46^b^	14.05^b^	0.796	0.009	0.518	0.003
CAT, U/mg protein	9.19	9.10	9.02	9.65	9.07	8.95	0.646	0.929	0.837	0.645
MDA, nmol/mg protein	0.94^a^	0.91^a^	0.93^a^	0.79^b^	0.90^a^	0.91^a^	0.021	< 0.001	0.016	< 0.001

CKT, *Caragana korshinskii* tannin; SOD, superoxide dismutase; GSH-Px, glutathione peroxidase; CAT, catalase; MDA, malondialdehyde; ^a,b^means with different letters differ significantly (*P* < 0.05); SEM, standard error of mean.

### 3.9 Effects of CKT on tight junction protein-related genes expression of jejunum tissues in mice

**Figure 7:**
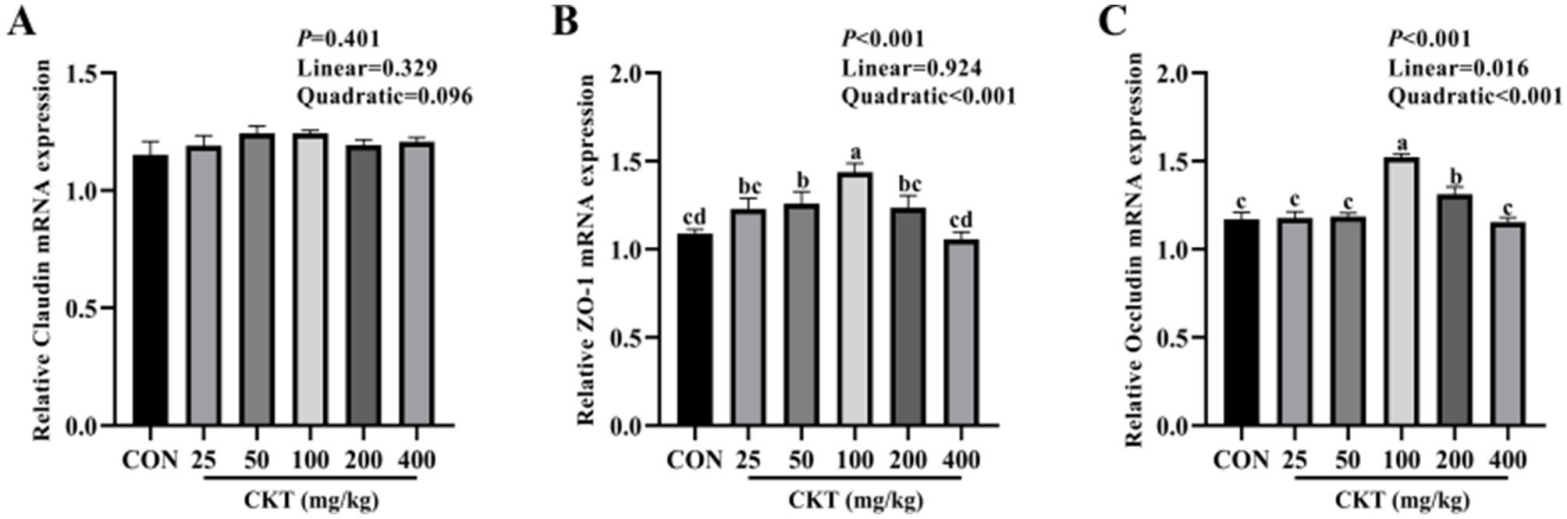
Effect of dietary supplementation with CKT on tight junction protein-related genes expression of jejunum tissues in mice. CKT, *Caragana korshinskii* tannin. **(A)** Relative Claudin mRNA expression, **(B)** Relative ZO-1 mRNA expression, **(C)** Relative Occludin mRNA expression.

As showed in Figure 7, with the increase of CKT concentration, the gene expression of ZO-1 and Occludin were increased quadratically (*P*_quadratic_ < 0.05), and the gene expression of Occludin were increased linearly (*P*_linear_ < 0.05), with the highest value at 100 mg/kg CKT (*P* < 0.05). Compared with the control group, the 50 mg/kg and 100 mg/kg CKT treatment significantly increased (*P* < 0.05) the mRNA expression level of ZO-1 in jejunum (Figure 7B). The 100 mg/kg and 200 mg/kg CKT treatment significantly increased (*P* < 0.05) the mRNA expression level of occludin in jejunum compared with the control group (Figure 7C). No significant different in the mRNA expression of claudin was observed between mice in CON group and CKT administration group (*P* > 0.05) (Figure 7A).

### 3.10 Effects of CKT on tight junction protein expression of jejunum tissues in mice

**Figure 8:**
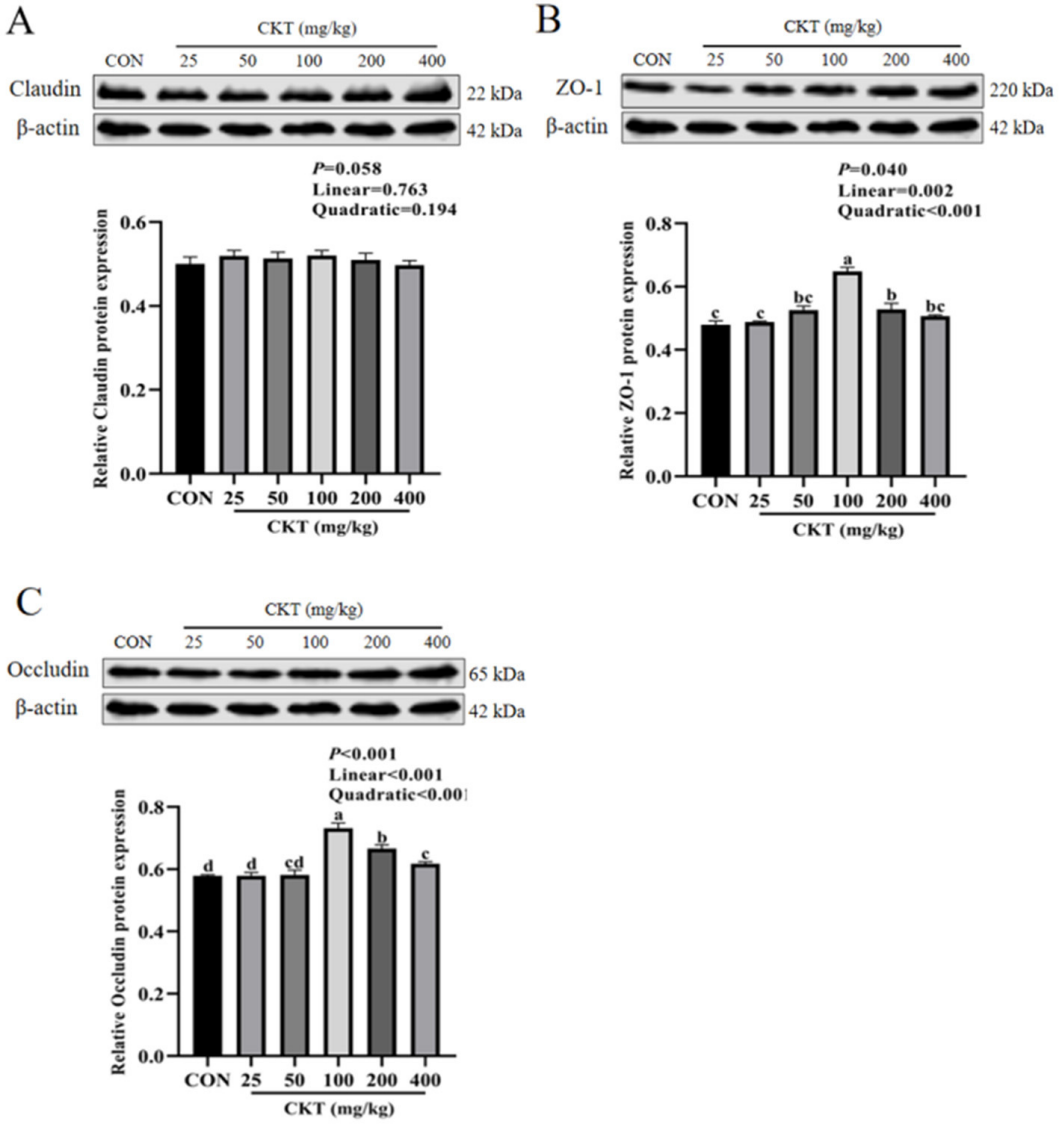
Effect of dietary supplementation with CKT on tight junction protein expression of jejunum tissues in mice. CKT, *Caragana korshinskii* tannin. **(A)** Relative Claudin protein expression, **(B)** Relative ZO-1 protein expression, **(C)** Relative Occludin protein expression.

The western blot results indicated that, with the increase of CKT concentration, both the protein expression of ZO-1 and occludin were increased linearly (*P*_linear_ < 0.05) and quadratically (*P*_quadratic_ < 0.05), with the highest value at 100 mg/kg CKT (*P* < 0.05). In addition, the expression of ZO-1 protein and occludin protein were markedly upregulated (*P* < 0.05) in the jejunum of mice in the 100 mg/kg CKT group compared to those in the CON and other CKT administration groups (Figures 8B, C). The mice in the CKT administration groups showed no significant regulation (*P* > 0.05) in the expression of the claudin protein compared to the CON group (Figure 8A).

## 4 Discussion

In this study, organic solvent extraction was successfully used to extract CT from the CK. RSM was employed to optimize extraction conditions. The optimum extraction conditions of CKT were as follows: extraction temperature of 52°C, extraction time of 95 min, liquid-solid ratio of 20:1 and acetone volume fraction of 62%. Under these conditions, the contents of CKT were 5.34%. While there is limited research specifically on the extraction of CKT, numerous studies have been conducted using similar materials. Sousa and Brito (35) studied the extraction of cashew tree bark CT and drew a conclusion that the best extraction conditions were extraction time 100 min, extraction temperature 100°C and L/S ratio of 30:1, respectively. Liu et al. (36) worked on astringent persimmon tannin and found that, at fixed ultrasonic temperature of 55°C, the tannin yield reached 33.96 mg/g under the optimal conditions: extraction time 100 min, L/S ratio of 21.1:1, and ultrasonic power of 207 W. they also found that persimmon tannin could be explored as a natural antioxidant food ingredient (36). The proanthocyanidins or CT feature a central heterocyclic ring connected to two phenolic rings via ether bonds (39). The optimum extraction process of tannin from different plants are different, and the extraction conditions are very important for tannin activity. Therefore, the follow-up research on tannin extraction process of CKT should still improve its yield under the condition of guaranteeing its activity. Our study showed that CKT obtained by the optimum extraction process had significant antioxidant activity *in vitro*. It has been demonstrated that *Moringa oleifera L*. leaves polyphenol with antioxidant effects are related to the structural polyphenol with molecular weights of 500–3,000 kDa, consisting mainly of gallic acid, orientin, quercetin, kaempferol, and catechin (40). Grape seeds condensed tannin exerts anti-oxidant effects mainly due to the presence of interflavan linkages, ester bonds, and the molecular structure with polyols as the core (41). From what has been discussed above, we may reasonably arrive at the conclusion that the biological function of tannin is closely related to its molecular weight and molecular structure. In our study, the CKT has a typical polyphenol peak with a molecular weight of 8.662 kDa and is composed primarily of epigallocatechin: catechin: epigallocatechin gallate: epicatechin: gallocatechin: epicatechin-3-o-gallate: catechin gallate = 1:8.88:2.65:1.55:1.92:0.49:0.14. It has been reported that catechin exhibited good *in vitro* antioxidant capacity (42, 43). This may be the reason that CKT has strong antioxidant capacity. TA has been shown to regulate ROS by chelating ferrous ions, helping to mitigate oxidative stress induced by iron overload in the liver (42). Our hypothesis is that CKT's pharmacological properties are also related to its structural properties, thus further research is required into the relationship between structure and activity. Additionally, to assess its safety and efficacy in functional foods or medicines, further studies using animal models are essential for *in vivo* evaluation.

Growth performance is a key indicator of animal development, and enhancing it can significantly boost economic benefits. While plant tannins were once deemed anti-nutritional due to their ability to bind proteins, starches, and digestive enzymes (44, 45). Recent research suggests that appropriate levels of tannins can actually improve growth, antioxidant function, and immune response in animals (46, 47). The variability in responses to dietary tannins across studies may stem from factors such as polymerization levels, purity, dosage, and source. Notably, in this study, the positive effects on growth performance were observed at doses of 25 mg/kg BW, 50 mg/kg BW, and 100 mg/kg BW of CKT, supporting previous findings that small quantities of suitable tannins may not exhibit anti-nutritional effects (48). Thus, our study determined the safe dose of 25 mg/kg BW to 100 mg/kg BW of CKT in mice experiment.

DAO serves as a marker enzyme in the epithelial cells of mammalian intestinal villi, while D-LA is a metabolic end product produced by intestinal bacteria (49). When the intestinal mucosal barrier is compromised, both DAO and D-LA are released into the bloodstream, indicating damage to the barrier (50). In this study, both D-lactic acid levels and DAO activities were significantly decreased by the addition of 100 mg/kg CKT. Similarly, previous literature showed that dietary supplementation of 0.2% and 1.0% TA resulted in a lower serum D-lactic acid levels and DAO activity in weaned piglets (2). This indicates that CKT improved intestinal permeability and ultimately protected intestinal barrier function. However, the specific mechanisms behind these improvements require further investigation.

The preservation of intestinal morphological structures is essential for maintaining normal intestinal function and overall gut health (2). In the present study, we found that 25, 50, 100, and 200 mg/kg CKT groups had significantly increased jejunum VH and VH:CD of mice compared with control group but the group given 400 mg/kg CKT did not differ in VH and VH:CD from the control. This effect could be attributed to the small amounts of CKT, which may have reduced the comparative aggression of this substance on the digestive mucosa, resulting in decreased mucus hypersecretion. Additionally, CKT appears to stimulate enterocyte proliferation in the middle crypt zone of the jejunum, contributing to enhanced intestinal health and function (51). However, large dose tannin-gavage mice exhibited epithelial degeneration and ulceration in the jejunum (52). These favorable alterations in intestinal morphology may also stem from the prebiotic function of tannins, which serve as substrates for intestinal microflora, stimulating fermentation and increasing short-chain fatty acid production. These short-chain fatty acids promote the differentiation and proliferation of intestinal epithelial cells. Furthermore, other researchers have suggested a positive association between intestinal morphology and antioxidant capacity in both broilers and mice (13, 53), similar conclusion was seen in our study as well.

Piglets suffering from weaning stress produce excessive amounts of ROS, which disrupts the balance between the oxidation system and the antioxidant system, causing oxidative stress and tissue damage, including damage to the intestinal barrier. Therefore, we speculate that oxidative stress inhibition may be an effective strategy to repair intestinal barrier damage. The SOD is one of the enzymes widely involved in oxidative stress, which can catalyze the conversion of superoxide to oxygen and H_2_O_2_ (54). The GSH-Px is an important antioxidant enzyme in the body and supports the integrity of cell membranes by catalyzing hydrogen peroxide decomposition *in vivo*. The MDA, is a marker of oxidative stress and is considered as an indicator to reflect the degree of cell damage and lipid peroxidation (55). Faraha et al. added plant tannin to a broiler diet to reduce the content of MDA in muscle (56). Dietary grape seed procyanidins enhance SOD and GSH-Px activities while reducing MDA levels in the jejunal and ileal mucosa of LPS-challenged chicks, thereby mitigating LPS-induced oxidative stress (57). Studies based on colonic epithelial cells from the monogastric animals found that Granny Smith appleprocyanidin extract upregulates the expression of the GSH-Px and SOD mRNA, and reduces oxidative stress and inflammatory response (58). These findings strongly support that tannins play a crucial role in enhancing antioxidant capacity through regulating the activity of antioxidant enzymes. In this study, we found that adding 100 mg/kg BW CKT resulted in higher activity and mRNA expression of SOD and GSH-Px, lower concentration of MDA in mouse jejunum tissues than in the control. One potential explanation for the antioxidant impact is the polyphenolic of tannin, its relatively hydrophobic “core” and hydrophilic “shell” are the features responsible for its antioxidant action (59). Exogenous antioxidants can aid in restoring oxidative balance and maintaining intestinal mucosa health in animals. Consequently, the growth-promoting effects of CKT might be attributed to its antioxidant properties and its protective role against intestinal dysfunction.

Intestinal epithelial cells are a major component of the intestinal epithelium, which are jointed by tight junctions (occludin, zo-1, and claudin) to form the epithelial barrier (60). Plant bioactive compounds play a crucial role in improving the integrity and function of the gastrointestinal tract by regulating tight junction protein expression and increasing intestinal antioxidant capacity. Previous study found that 2.5 mg/kg and 5 mg/kg tannic acid significantly upregulated the mRNA expression of zo-1 in mice (61). Our study established that 100 mg/kg CKT promoted the gene expression of ZO-1 and occludin in jejunum, but CKT pretreated mice failed to regulate the gene expression of claudin. This might be due to the increase of the mRNA expression of ZO-1 and occludin in CKT pretreated mice as an antioxidative response of the body to improve intestinal epithelial barrier. Moreover, according to a study conducted on Wistar Furth rats, grape seed extract does not improve claudin-1 expression in the cecum or colon, possibly because of differences in microbial distribution, resulting in different metabolite profiles of tannin (62). These results suggested that CKT could improve the intestinal barrier function in mice by increasing the expression of tight junction protein.

## 5 Conclusions

**Figure 9:**
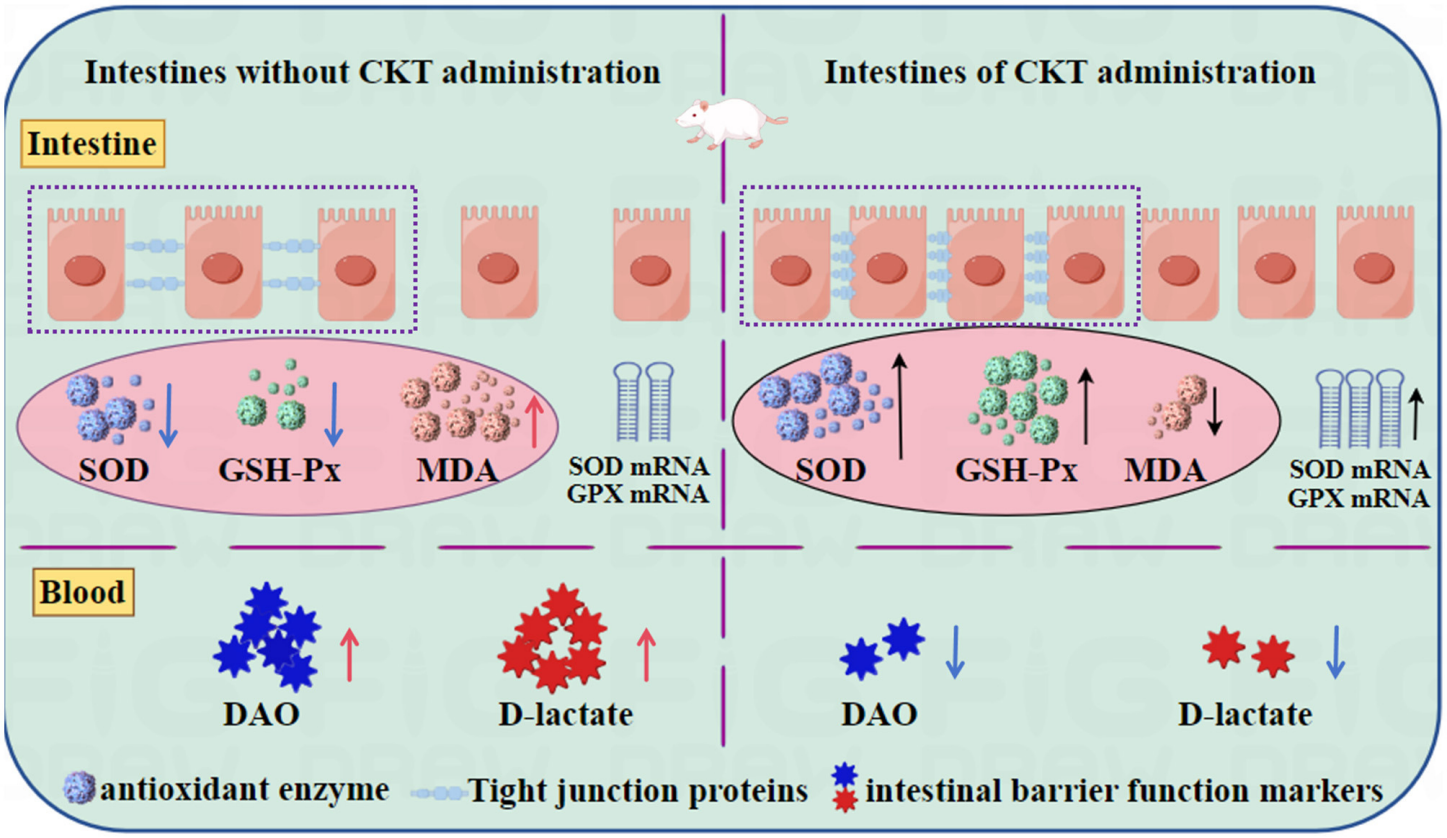
CKT improves intestinal barrier function and maintains intestinal homeostasis in mice. CKT, *Caragana korshinskii* tannin.

The optimum extraction conditions of CT from CKT were as follows: extraction temperature of 52°C, extraction time of 95 min, liquid-solid ratio of 20:1 and acetone volume fraction of 62%. Under these conditions, the experimental yield was obtained as 5.34%. The CKT has a typical polyphenol peak with a molecular weight of 8.662 kDa and is composed of epigallocatechin: catechin: epigallocatechin gallate: epicatechin: gallocatechin: epicatechin-3-o-gallate: catechin gallate = 1:8.88:2.65:1.55:1.92:0.49:0.14. Additionally, CKT exhibited positive radical scavenging activities against DPPH radical, ABTS radical, hydroxyl radicals, and metal chelating ability *in vitro*. CKT could promote growth performance of mice, and improve the physiological status, physical and biochemical barrier function of jejunum by modulating antioxidant enzyme, tight junction proteins and markers of intestinal barrier function (Figure 9).

## Data Availability

The original contributions presented in the study are included in the article/supplementary material, further inquiries can be directed to the corresponding authors.
